# Deep vein thrombosis of lower limbs in patients with COVID-19

**DOI:** 10.1590/1677-5449.202300272

**Published:** 2023-11-27

**Authors:** Orlando Adas Saliba, Ana Flávia de Jesus Alves, Camila Matarazzo, Gabriela Teixeira Gonçalves, Marcone Lima Sobreira

**Affiliations:** 1 Universidade Estadual Paulista “Júlio de Mesquita Filho” - UNESP, Botucatu, SP, Brasil.; 2 Centro Universitário Católico Auxilium - UniSALESIANO, Araçatuba, SP, Brasil.

**Keywords:** anticoagulant, SARS-CoV-2, thromboembolism

## Abstract

As knowledge has accumulated, COVID-19 has come to be considered a disease of the respiratory system that can also cause multisystemic involvement. This study analyzed the prevalence of deep venous thrombosis (DVT) in the lower limbs of patients with COVID-19 by conducting an integrative review of the literature published from 2019 to 2022. The procedures involved in article selection were identification of keywords, definition of the search strategy, consultation of databases, and exclusion of duplicate articles and others that did not meet the review objectives. Exclusion of articles was based on the following exclusion criteria: articles on arterial vascular complications involving the lower limbs, laboratory experiments, cases reports describing venous and arterial complications involving other sites, and articles unrelated to the outcome of interest: DVT. A total of 284 articles were identified, 42 of which were included. There was considerable variability in the prevalence of DVT among patients with COVID-19 (range: 0.43 to 60.87%). The findings suggest that occurrence of DVT in patients with COVID-19 is associated with disease severity.

## INTRODUCTION

Coronaviruses (CoVs) are single-stranded ribonucleic acid (RNA) viruses that cause diseases in humans and animals.^1^ In December of 2019, a new coronavirus strain was identified in patients presenting with pneumonia of unknown etiology and was named severe acute respiratory syndrome coronavirus 2 (SARS-CoV-2) by the International Committee on Taxonomy of Viruses (ICTV).^1^ The same month, the first case of coronavirus disease 2019 (COVID-19) was confirmed in the city of Wuhan, in China.^2^ The disease caused a global outbreak that was designated as a pandemic by the World Health Organization (WHO) on March 11, 2020.^3^

This pandemic spread rapidly, with more than 199 million confirmed cases and more than 4 million deaths worldwide during 2021,^4^ demonstrating high rates of contagion, morbidity, mortality, and lethality, provoking a major overload of health care systems.^5^

Transmission of COVID-19 occurs person-to-person by direct contact, by aerial transmission in aerosols, and during medical procedures.^6^ The incubation period of SARS-CoV-2 can be considered as from 2 to 14 days, with a mean period of 5 days,^7^ and it is diagnosed by testing nasopharyngeal swab samples with reverse transcriptase followed by polymerase chain reaction (RT-PCR).^8^

Around 80% of confirmed cases of COVID-19 are asymptomatic or mildly symptomatic;^9^ 15% progress to a more severe form; and 5% develop acute respiratory distress syndrome (ARDS) and require ventilatory support.^7^

Patients considered to have mild to moderate severity exhibit the following clinical manifestations: fever (88.7%), coughing (67.8%), tiredness (38.1%), productive expectoration (33.4%), dyspnea (18.7%), sore throat (13.9%), and headache (13.6%).^1^

Severe infection is characterized by uncontrolled multisystemic inflammatory and immune response, with cardiovascular, respiratory, neurological, intestinal, hepatic, pancreatic, renal, cutaneous, and hematological involvement. This response is mediated by direct viral action, with endothelial dysfunction, inflammation, and thrombosis of the microcirculation of organs,^10^ and may induce sepsis, ARDS, bilateral interstitial pneumonia, multiple organ failure, and disseminated intravascular coagulation (DIC), and can lead to fatal outcomes.^11^ The activation of blood coagulation provokes a prothrombotic state with significant elevation of fibrin, of fibrin degradation products (including D-dimer [DD]) and fibrinogen. The resulting state is known as COVID-19 associated coagulopathy (CAC). The pathophysiology of CAC includes cytokine storm, causing activation of endothelial cells and pulmonary microvasculature injury, causing local microthrombosis, and a hypercoagulable state that can result in thrombosis of large vessels.^2^

From this perspective, the procoagulatory nature of COVID-19, combined with other risk factors, such as immobility, mechanical ventilation, and infection, predisposes patients to thromboembolic complications,^7^ such as deep venous thrombosis (DVT), pulmonary embolism (PE), arterial thrombosis, pulmonary thrombosis (PT),^12^ unusual thrombosis of central lines or arterial catheters, and premature thromboses of extrarenal hemodialysis filters and extracorporeal membrane oxygenation (ECMO) cannulae.^7^

Considering the importance of the vascular involvement related to COVID-19 and the need for data to support decision making on the most effective forms of thromboprophylaxis, it is essential to discuss the impact of these complications and the different treatment approaches, justifying a wide-ranging review of the literature.

In view of the above, the objective of this study was to analyze the prevalence of DVT in the lower limbs of patients with COVID-19.

## METHODS

This is an integrative review of the literature on COVID-19-related DVT in lower limbs, written up in accordance with the PRISMA protocol.^13^

The procedures employed to identify and select scientific output were based on consultations of the SciELO, PubMed, Cochrane, Scopus, Web of Science, and LILACS databases, considering publications from December 2019, when the pandemic started, to September 2022.

Relevant keywords were selected and the following search strategy was defined: COVID19 OR COVID-19 Viral Disease OR 2019-nCoV Disease OR Coronavirus 2019 Disease OR 2019-nCoV Coronavirus Disease OR Coronavirus-19 Disease OR Novel Coronavirus 2019 Disease OR COVID-19 Virus Disease OR Wuhan Coronavirus Pneumonia Epidemic OR Wuhan 2019-2020 Coronavirus Pneumonia Epidemic OR Coronavirus from Wuhan Pneumonia Epidemic OR Coronavirus from Wuhan 2019-2020 Pneumonia Epidemic OR Novel Coronavirus 2019-2020 Pneumonia Epidemic OR Wuhan Coronavirus Epidemic OR Coronavirus from Wuhan Epidemic OR Novel Coronavirus 2019 Epidemic OR 2019-nCoV Epidemic OR Wuhan Coronavirus Epidemic OR Coronavirus from Wuhan Epidemic OR Novel Coronavirus 2019 Epidemic OR Wuhan Coronavirus Pneumonia Fever OR COVID-19 Viral Infection OR Infection by the 2019-nCoV Coronavirus OR Infection by the Wuhan Coronavirus OR Infection by the SARS-CoV-2 OR 2019-nCoV Infection OR 2019-nCoV Coronavirus Infection OR Wuhan Coronavirus Infection OR Novel Coronavirus 2019 Infection OR SARS Coronavirus 2 Infection OR SARS-CoV-2 Infection OR COVID-19 Virus Infection OR SARS-CoV-2 Infections OR COVID-19 Pandemic OR COVID-19 Pandemics OR Wuhan Seafood Market Pneumonia OR Wuhan Coronavirus Pneumonia OR Novel Coronavirus 2019-2020 Pneumonia OR Wuhan Coronavirus Outbreak OR 2019-2020 Chinese Pneumonia Outbreak OR Pneumonia Outbreak in China 2019-2020 OR 2019-nCoV Coronavirus Outbreak OR Wuhan Coronavirus Outbreak OR Wuhan Coronavirus 2019-2020 Outbreak OR Novel Coronavirus 2019 Outbreak OR 2019-nCoV Outbreak OR 2019-nCoV Coronavirus Outbreak OR Wuhan Coronavirus Outbreak OR Wuhan Coronavirus 2019-2020 Outbreak OR Novel Coronavirus 2019 Outbreak OR COVID-19 Virosis) AND (Deep Venous Thrombosis lower limbs OR Deep Venous Thrombosis lower limb OR Deep Vein Thrombosis lower limbs OR Deep Vein Thrombosis lower limb OR Thrombosis of lower limbs deep veins OR Thrombosis of lower limb deep veins OR Deep Vein Thrombosis lower extremities OR Deep Vein Thrombosis lower extremity).

The review included cross-sectional, case-control, and cohort studies that were directly related to the subject of interest. The first step in analysis of the articles was reading the titles and abstracts to classify them by study type and eliminate duplicated publications. The full texts of articles were then read to identify the characteristics, objectives, and results of each study. Data were extracted from each article and input to a dedicated spreadsheet containing title, authors, year of publication, country in which the study was conducted, periodical, objective, type of study, sample, tests used for diagnosis, main results, and evidence level. An analysis was conducted to determine the degree of evidence of each article using the SORT (Strength of Recommendation Taxonomy) protocol. This classification stratifies evidence levels as: A) consistent and good quality patient-oriented evidence; B) inconsistent or limited quality patient-oriented evidence; or C) consensus, usual practice, opinion, disease-oriented evidence, or case series for studies of diagnosis, treatment, prevention, or screening.

The review included articles related to the primary outcome of interest - symptomatic and asymptomatic DVT - and also cases diagnosed incidentally or systematically in the lower limbs of patients with confirmed COVID-19. Case reports and articles on arterial and venous vascular complications located in other anatomic sites were excluded. A total of 284 articles were identified, 42 of which were analyzed.

**Figure 1:**
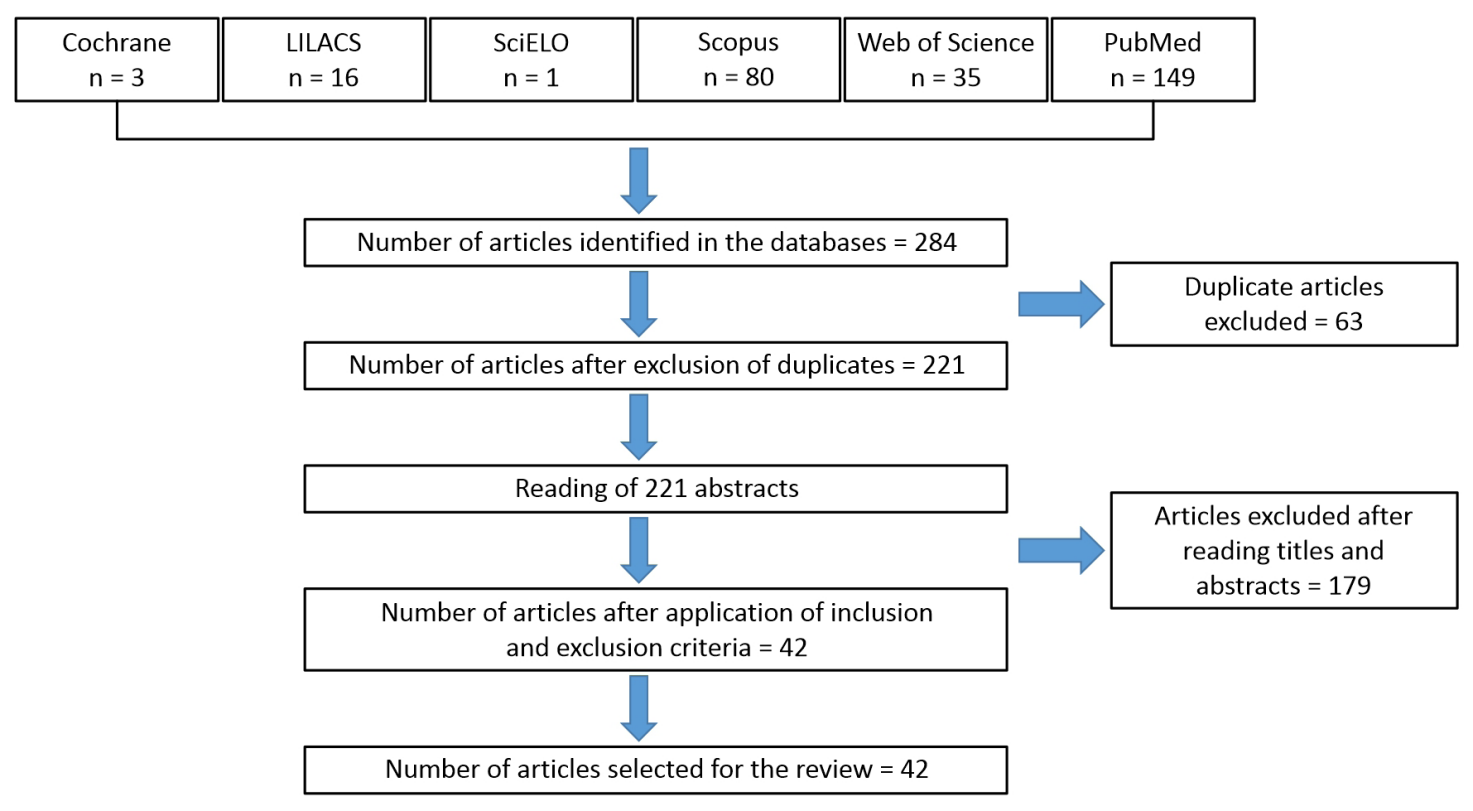
Search strategy used for literature review of lower limb deep venous thrombosis in patients with COVID-19.

Figure 1 illustrates the procedures employed and the process for selection of articles from the databases.

## RESULTS

Table 1 lists the data extracted from each of the 42 scientific articles selected for the review, including title, authors, year of publication, country in which the study was conducted, periodical, objective, study type, sample, tests used for diagnosis of DVT, and main results on disease prevalence. The studies included (total n = 42) were conducted Italy (n = 12), the United States (n = 8), France (n = 6), China (n = 5), Spain (n = 4), Brazil (n = 2), Argentina (n = 1), Croatia (n = 1), Egypt (n = 1), Sweden (n = 1), and Switzerland (n = 1). It was observed that the majority of the articles were cohort studies. Ultrasound was the supplementary test most often used to diagnose DVT in the lower limbs. There was major variability in the results for prevalence (range: 0.43 to 60.87%). The majority of articles were classified at evidence level A (n = 19).

**Table 1:** Studies selected for review of deep venous thrombosis in the lower limbs of patients with COVID-19.

**NO.**	**Title**	**Author**	**Year**	**Country**	**Periodical**	**Objective**	**Study type**	**Sample size**	**Examination**	**Results** **(number of patients with DVT)**	**Evidence level**
1	Thrombosis and bleeding after implementation of an intermediate-dose prophylactic anticoagulation protocol in ICU patients with COVID-19: a multicenter screening study	Al-Abani et al.^14^	2022	Sweden	Journal of Intensive Care Medicine	To study the prevalence of venous thromboembolism and bleeding in patients with critical COVID-19 after intermediate-dose prophylactic anticoagulation.	Prospective observational study	100 patients	Two-region compression ultrasound of the lower extremities, including the common femoral, femoral, and popliteal veins, with the patient in the supine position	20/100 patients (20,00%), including 19 asymptomatic cases	A
2	Venous Doppler ultrasound in critically ill COVID-19 patients: game changer in anticoagulation therapy	Alfageme et al.^15^	2020	Spain	Ultrasound Journal	To evaluate the prevalence of asymptomatic DVT in lower limbs in critically ill COVID-19 patients with severe respiratory failure and high levels of D-dimer.	Observational, descriptive, and retrospective study	23 patients	Doppler ultrasound	14/23 patients (60.87%), 5 in proximal venous territories and 9 in infrapopliteal veins	C
3	COVID-19 and venous thromboembolism in intensive care or medical ward	Avruscio et al.^16^	2020	Italy	Clinical and Translational Science	To evaluate the incidence of venous thromboembolism in patients with COVID‐19 admitted to both intensive care units and medical wards.	Observational cohort study	85 patients, 44 admitted to medical wards and 41, to intensive care units	Systematic bilateral color Doppler ultrasonography	20/85 patients (23.53%), 6 patients admitted to medical wards and 14 in intensive care units	B
4	Incidence of deep venous thrombosis in COVID-19 hospitalized patients during the first peak of the Italian outbreak	Baccellieri et al.^17^	2021	Italy	Phlebology	To assess the incidence of deep vein thrombosis in COVID-19 patients admitted to a tertiary hospital.	Retrospective observational study	200 patients	Vascular ultrasonography in all patients	29/200 patients (14.50%), 5/40 (12.50%) patients in the intensive care unit and 24/160 (15,00%) patients not admitted to the intensive care unit	B
5	Deep vein thrombosis in hospitalized patients with coronavirus disease 2019	Chang et al.^18^	2021	United States	Journal of Vascular Surgery: Venous and Lymphatic Disorders	To assess experience with deep venous thrombosis in patients with COVID-19.	Retrospective, single-center, cohort analysis study	188 patients	Duplex ultrasound performed by accredited vascular technicians and formally read and reviewed by vascular surgeons	53/188 patients (28.20%).	C
6	Pulmonary embolism or thrombosis in ARDS COVID-19 patients: a French monocenter retrospective study	Contou et al.^19^	2020	France	PLoS One	To assess the clinical features and the outcomes of patients with acute respiratory distress syndrome caused by COVID-19 diagnosed with pulmonary embolism during intensive care unit stay.	Retrospective observational study	16 patients	Doppler venous ultrasonography of the lower limbs of patients who exhibited pulmonary embolism	3/16 patients (19,00%)	A
7	Comparison of deep vein thrombosis risks in acute respiratory distress syndrome caused by COVID-19 and bacterial pneumonia: a retrospective cohort study	Cui et al.^20^	2022	China	Thrombosis Journal	To compare the incidence and risks of deep venous thrombosis between patients with acute respiratory distress syndrome caused by COVID-19 and by bacterial pneumonia.	Retrospective cohort study	105 patients	Lower limb ultrasonography with venous compression maneuver, including bilateral common femoral, deep and superficial femoral, popliteal, anterior tibial, posterior tibial, fibular, and calf veins	60/105 patients (57.10%)	B
8	Is venous thromboembolism a predictable marker in older patients with COVID-19 infection? A single-center observational study	De Giorgi et al.^21^	2021	Italy	The Journal of Infection in Developing Countries	To analyze if laboratory exams, risk assessment scores, and comorbidity risk scores were useful in predicting deep venous thrombosis in SARS-CoV-2 patients admitted to internal medicine wards.	Observational cohort study	49 patients	Color Doppler venous ultrasonography of the lower limbs	10/49 patients (20.40%)	A
9	Imaging of COVID-19 vasculopathy from head to toe: Egyptian collective experience after 2 years of the pandemic	Fathy et al.^22^	2022	Egypt	Egyptian Journal of Radiology and Nuclear Medicine	To provide the Egyptian experience about the COVID-19 vasculopathy during the past two years of the pandemic and to collectively include the different modalities and imaging techniques for the diagnosis of cerebrovascular, pulmonary, gastrointestinal, and peripheral arterial vascular complications..	Retrospective cohort study	282 patients	Duplex ultrasound examinations	39/282 patients (13.80%)	A
10	COVID-19: persistence of symptoms and lung alterations after 3-6 months from hospital discharge	Fortini et al.^23^	2021	Italy	Infection	To evaluate the results of a follow-up program for patients discharged from a nonintensive COVID-19 ward.	Prospective observational study	59 patients	Ultrasonography with compression maneuver	1/59 patients (1.70%)	A
11	Point of care ultrasound (POCUS) in diagnosis of proximal deep vein thrombosis among COVID-19 hospitalized patients with a high rate of low molecular weight heparin prophylaxis	García-Ceberino et al.^12^	2021	Spain	Medicina Clinica	To determine proximal deep venous thrombosis prevalence with point of care ultrasound screening among hospitalized COVID-19 patients.	Retrospective observational study	87 patients	Lower limb ultrasonography	4/87 patients (4.60%)	C
12	Probative value of the D-dimer assay for diagnosis of deep venous thrombosis in the coronavirus disease 2019 syndrome	Gibson et al.^24^	2020	United States	Critical Care Medicine	To describe the predictive utility of the D-dimer assay among patients with the coronavirus disease 2019 syndrome for unprovoked lower extremity deep venous thrombosis.	Prospective observational study	72 patients	Duplex lower limb ultrasonography	12/72 patients (16.70%)	A
13	SARS-CoV-2 and finding of vein thrombosis: can IMPROVE and IMPROVEDD scores predict COVID-19 outcomes?	Greco et al.^25^	2021	Italy	European Review for Medical and Pharmacological Sciences	To analyze the relationship between validated predictive scores for venous thromboembolism such as IMPROVE and IMPROVEDD and intensification of care, in-hospital mortality rate, and 30-days mortality rate.	Retrospective observational study	51 patients	Color Doppler venous ultrasonography of the lower limbs	11/51 patients (21.60%)	A
14	Systematic duplex ultrasound screening in conventional units for COVID-19 patients with follow-up of 5 days	Hamadé et al.^26^	2021	France	Journal of Vascular Surgery: Venous and Lymphatic Disorders	To evaluate the prevalence of deep venous thrombosis of lower limbs through ultrasonography in patients infected with COVID-19.	Retrospective cohort study	72 patients	Venous duplex ultrasound of the lower limbs	12/72 patients (16.70%)	C
15	Efficacy and safety of sonographer discretion to terminate a venous duplex ultrasound for diagnosis of deep vein thrombosis in coronavirus disease 2019 patients	Ho et al.^27^	2022	United States	Journal of Vascular Surgery: Venous and Lymphatic Disorders	To evaluate the efficacy of a modified COVID-19 venous duplex ultrasound protocol to reduce sonographer exposure to COVID-19 patients.	Retrospective cohort study	168 patients	Venous duplex ultrasound of the lower limbs	44/168 patients (27.50%)	A
16	Deep venous thrombosis incidence in patients with COVID-19 acute respiratory distress syndrome, under intermediate dose of chemical thromboprophylaxis	Hunter et al.^28^	2022	Argentina	Medicina (Buenos Aires)	To assess the incidence of deep venous thrombosis in patients with severe pneumonia due to COVID-19, requiring mechanical ventilation and on intermediate chemical thromboprophylaxis doses.	Prospective cohort study	46 patients	Lower limb Doppler ultrasonography	3/46 patients (6.52%)	A
17	Deep vein thrombosis in COVID-19 patients in general wards: prevalence and association with clinical and laboratory variables	Ierardi et al.^29^	2021	Italy	La Radiologia Medica	To report the prevalence of deep venous thrombosis in the lower limbs of COVID-19 patients in general wards.	Cross-sectional study	263 patients	Duplex lower limb ultrasonography	67/263 patients (25.50%)	C
18	Examining D-dimer and empiric anti-coagulation in COVID-19-related thrombosis	Johnson et al.^30^	2022	United States	The Cureus Journal of Medical Science	To elucidate the relationship between macro/microvascular thrombosis, D-dimer levels, and empiric anticoagulation in COVID-19.	Prospective observational study	52 patients	Venous duplex ultrasound of the lower limbs	1/52 patients (1.90%)	A
19	Thromboembolic events and role of point of care ultrasound in hospitalized COVID-19 patients needing intensive care unit admission	Kapoor et al.^2^	2021	United States	Journal of Intensive Care Medicine	To investigate the cumulative incidence of thromboembolic events in COVID-19 patients in intensive care units and assess the utility of point of care ultrasound to diagnose lower extremity deep venous thrombosis.	Prospective observational study	107 patients	Lower limb ultrasonography	21/107 patients (19.63%)	C
20	Screening for deep vein thrombosis in persons with COVID-19 upon admission to an inpatient rehabilitation hospital	Kirshblum et al.^3^	2021	United States	American Journal of Physical Medicine & Rehabilitation	To determine the prevalence of deep venous thrombosis detected by duplex screening and risk factors associated with deep venous thrombosis in patients with COVID-19.	Retrospective observational study	113 patients	Duplex ultrasonography screening of both lower limbs	25/113 patients (22,00%)	A
21	Clinical characteristics of acute lower extremity deep venous thrombosis diagnosed by duplex in patients hospitalized for coronavirus disease 2019	Koleilat et al.^31^	2021	United States	Journal of Vascular Surgery: Venous and Lymphatic Disorders	To characterize patients with deep venous thrombosis identified after admission for COVID-19.	Retrospective case-control study	135 patients	Venous duplex ultrasound of the lower limbs	18/135 patients (13.30%)	C
22	Systematic screening for deep vein thrombosis in critically ill in patients with COVID-19: impact on the incidence of venous thromboembolism	Lapébie et al.^32^	2021	France	Frontiers in Medicine	To compare the incidence of venous thromboembolism of two different methods for lower extremity deep vein thrombosis diagnosis.	Prospective cohort study	27 patients	Venous ultrasonography of the lower limbs with complete compression maneuver	7/27 patients (25.90%)	B
23	Venous thromboembolism in critically Ill patients with COVID-19: results of a screening study for deep vein thrombosis	Longchamp et al.^33^	2020	Switzerland	Research and Practice in Thrombosis and Haemostasis	To determine the prevalence of venous thromboembolism in critically ill patients with COVID-19, using lower limb venous ultrasonography screening.	Prospective observational study	25 patients	Lower limb ultrasound with venous compression maneuver	6/25 patients (24,00%)	C
24	Asymptomatic deep vein thromboses in prolonged hospitalized COVID-19 patients	Lucijanic et al.^34^	2021	Croatia	Wiener klinische Wochenschrift	To assess the prevalence of deep venous thrombosis among prolonged hospitalized COVID-19 patients without clinical suspicion of deep venous thrombosis and to investigate potential predictors.	Prospective observational study	102 patients	Bilateral duplex ultrasonography of the deep veins of the lower limbs	26/102 patients (25.50%)	A
25	Pulmonary thromboembolism in hospitalised COVID-19 patients at moderate to high risk by Wells score: a report from Lombardy, Italy	Monfardini et al.^35^	2020	Italy	The British Journal of Radiology	To present a single-centre experience of CT pulmonary angiography for the assessment of hospitalized COVID-19 patients with moderate-to-high risk of pulmonary thromboembolism.	Retrospective observational study	33 patients	Doppler venous ultrasonography of the lower limbs	4/33 patients (12.12%)	C
26	Lower limb deep vein thrombosis in COVID-19 patients admitted to intermediate care respiratory units	Pancani et al.^36^	2021	Italy	Thrombosis Research	To investigate the incidence of lower limb deep vein thrombosis in patients admitted to medical wards and not requiring admission to an intensive care unit.	Prospective observational study	66 patients	Ultrasonography with compression maneuver	2/66 patients (3,00%)	C
27	Incidence of venous thromboembolism in patients with non-hematological cancer admitted for COVID-19 at a third-level hospital in Madrid	Paredes-Ruiz et al.^37^	2022	Spain	Journal of Thrombosis and Thrombolysis	To describe the incidence rate of venous thromboembolism in patients with non-hematological cancer who required hospitalization due to COVID-19.	Prospective observational study	11 patients	Lower limb Doppler ultrasonography	2/11 patients (18,00%)	C
28	Mortality and change in the prevalence of deep vein thrombosis associated with SARS-CoV-2 P.1 variant	Pereira de Godoy et al.^5^	2022	Brazil	Cureus	To determine the monthly and overall mortality rates by sex and age group in patients hospitalized with COVID-19 and the prevalence of deep vein thrombosis in those patients.	Case-control study	6,199 patients	Bilateral venous Doppler ultrasonography	254/6,199 patients (4.10%)	A
29	Incidence of deep vein thrombosis through an ultrasound surveillance protocol in patients with COVID-19 pneumonia in non-ICU setting: a multicenter prospective study	Pieralli et al.^38^	2021	Italy	PLoS One	To assess the incidence of deep vein thrombosis of the lower limbs in acutely ill patients with COVID-19 pneumonia admitted to non-intensive units.	Prospective cohort study	227 patients	Serial color-coded Doppler and compression ultrasonography	31/277 patients (13.70%)	C
30	Coagulation parameters and venous thromboembolism in patients with and without COVID-19 admitted to the Emergency Department for acute respiratory insufficiency	Pizzi et al.^39^	2020	Italy	Thrombosis Research	To compare coagulation parameters on admission between COVID-19 patients and non-COVID-19 patients with acute respiratory insufficiency and to describe venous thromboembolic events diagnosed.	Retrospective observational study	162 patients	Limited compression ultrasonography of the lower limbs.	3/162 patients (1.85%)	A
31	Combined use of Wells scores and D-dimer levels for the diagnosis of deep vein thrombosis and pulmonary embolism in COVID-19: a retrospective cohort study	Raj et al.^40^	2021	United States	Cureus	To assess the utility of Wells deep venous thrombosis and pulmonary embolism scores, and D-dimers in diagnosing deep venous thrombosis and pulmonary embolism in patients with COVID-19.	Retrospective cohort study	106 patients	Lower extremity duplex	35/106 patients (33,00%)	A
32	Rivaroxaban versus no anticoagulation for post-discharge thromboprophylaxis after hospitalisation for COVID-19 (MICHELLE): an open-label, multicentre, randomised, controlled trial	Ramacciotti et al.^41^	2022	Brazil	Lancet	To determine whether patients hospitalized with COVID-19 given prophylaxis with rivaroxaban (10 mg/day for 35 days after discharge) would have better clinical outcomes, including major and fatal thromboembolic events.	Multicenter, randomized and controlled clinical trial	252 patients	Bilateral venous Doppler ultrasound of the lower limbs	4/252 patients (1.59%)	A
33	Extremely high incidence of lower extremity deep venous thrombosis in 48 patients with severe COVID-19 in Wuhan	Ren et al.^42^	2020	China	Circulation	To analyze the prevalence of formation of thrombosis in patients with COVID-19.	Cross-sectional study	48 patients	Lower limb ultrasound with compression maneuver	41/48 patients (85.40%)	C
34	High prevalence of deep venous thrombosis in non-severe COVID-19 patients hospitalized for a neurovascular disease	Rouyer et al.^43^	2020	France	Cerebrovascular Diseases Extra	To report data obtained after systematic Doppler ultrasound scanning of lower limbs of non-severe COVID-19 patients.	Prospective observational study	13 patients	Doppler ultrasonography of the lower limbs	5/13 patients (38.46%)	C
35	Incidence of deep vein thrombosis among non-ICU patients hospitalized for COVID-19 despite pharmacological thromboprophylaxis	Santoliquido et al.^44^	2020	Italy	Journal of Thrombosis and Haemostasis	To determine the incidence of venous thromboembolism among non-intensive care unit patients hospitalized for COVID-19 who receive pharmacological prophylaxis.	Retrospective cohort study	84 patients	Ultrasonography with venous compression maneuver in the lower limbs	10/84 patients (11.90%)	A
36	Pulmonary thromboembolism in coronavirus disease 2019 patients undergoing thromboprophylaxis	Schiaffino et al.^45^	2021	Italy	Medicine (Baltimore)	To investigate the prevalence of pulmonary thromboembolism and its association with clinical variables in a cohort of hospitalized COVID-19 patients receiving low-molecular-weight heparin at prophylactic dosage.	Retrospective observational study	33 patients	Lower limb Doppler ultrasonography	1/33 patients (3,00%)	A
37	Duplex ultrasound screening for deep and superficial vein thrombosis in COVID-19 patients	Tung-Chen et al.^46^	2022	Spain	Journal of Ultrasound in Medicine	To determine the real incidence of deep or superficial venous thrombosis in patients with COVID-19.	Prospective observational study	233 patients	Bilateral duplex ultrasonography of the lower limbs	1/233 patients (0.43%)	C
38	Characteristics of deep vein thrombosis in the critically ill COVID-19 patient - an observational cohort study with Doppler ultrasound measurements	Voicu et al.^47^	2022	France	European Review for Medical and Pharmacological Sciences	To report deep venous thrombosis characteristics, vein diameters, and peak blood flow velocities in the common femoral veins of critically ill COVID-19 patients.	Prospective cohort study	55 patients	Lower limb Doppler ultrasonography	19/55 patients (35,00%)	C
39	Imbalance between procoagulant factors and natural coagulation inhibitors contributes to hypercoagulability in the critically ill COVID-19 patient: clinical implications	Voicu et al.^48^	2020	France	European Review for Medical and Pharmacological Sciences	To investigate the balance between procoagulant factors and natural coagulation inhibitors in the critically ill COVID-19 patient and to evaluate the usefulness of hemostasis parameters to identify patients at risk of venous thromboembolic event.	Prospective observational study	85 patients	Lower limb venous duplex ultrasound	35/85 patients (41.18%)	A
40	Analysis of risk factors for thromboembolic events in 88 patients with COVID-19 pneumonia in Wuhan, China: a retrospective descriptive report	Wang et al.^49^	2021	China	Medical Science Monitor	To analyze the risk factors and incidence of thrombosis in patients with confirmed COVID-19 pneumonia.	Retrospective observational study	88 patients	Doppler ultrasound	19/88 patients (21.59%)	B
41	Incidence and risk factors of deep vein thrombosis in hospitalized COVID-19 patients	Yu et al.^50^	2020	China	Clinical and Applied Thrombosis/Hemostasis	To evaluate the incidence rate and risk factors of deep venous thrombosis in patients with COVID-19.	Retrospective observational study	142 patients	Lower limb ultrasound with venous compression maneuver	50/142 patients (35.21%)	C
42	Deep vein thrombosis in hospitalized patients with COVID-19 in Wuhan, China: prevalence, risk factors, and outcome	Zhang et al.^51^	2020	China	Circulation	To investigate deep venous thrombosis in patients hospitalized with COVID-19.	Cross-sectional observational study	143 patients	Lower limb venous ultrasonography	66/143 patients (46.15%)	C

## DISCUSSION

This integrative review found that many different studies have been conducted in different parts of the world to investigate the signs and symptoms of COVID-19 and its systemic complications, such as DVT in the lower limbs. Clinical and epidemiological data are essential to guide planning and implementation of provision of health care for patients with these diseases.

It was observed that the majority of these studies were conducted in countries in Europe and America and just one study was conducted in Africa. In view of this, it is clearly important to conduct studies with different populations, considering the diversity of ethnicities, cultures, and social and economic conditions, with the objective of better understanding the multisystemic characteristics of the disease and identifying possible factors that could be associated with occurrence of DVT in the lower limbs of patients with COVID-19.

This review found that the incidence of cases of DVT in the lower limbs of patients with COVID-19 was investigated in different population groups, with mild, moderate, and severe forms of COVID-19, and it is possible that these factors have influenced the variability in the data reported by the studies analyzed.

A study conducted at the Wuhan Union Hospital, in China, with 88 patients admitted with confirmed COVID-19 pneumonia, 31 of which were critical cases, 33 severe cases, and 24 mild cases, identified DVT in the lower limbs of 19 patients (21.59%), 12 in critical patients and 7 in severe cases. This therefore suggests that occurrence of DVT in patients with COVID-19 may be related to disease severity, since it is more frequent in patients admitted to intensive care units in respiratory distress with respiratory rate ≥ 30 breaths/minute; peripheral oxy-hemoglobin saturation ≤ 93% at rest; ratio of partial pressure of oxygen/fraction of inspired oxygen ≤ 300 mmHg; respiratory failure requiring mechanical ventilation; shock; or failure of other organs.^16,50^

It is important to point out that, although the possible mechanisms and factors related to thromboembolic changes found in patients with COVID-19 have not been completely elucidated, the literature has demonstrated that formation of proinflammatory cytokines, induction of procoagulatory factors, and hemodynamic changes that predispose to ischemia and thrombosis can contribute to development of DVT in the lower limbs of these patients. Furthermore, changes that can be detected with laboratory tests, such as lymphopenia, neutrophilia, elevated prothrombin time, and elevated DD, have been observed in patients who develop venous thromboembolism.^5^

Recent studies have shown that patients hospitalized for COVID-19 are at high risk of development of thromboembolic events, and, consequently, there was a massive increase in requests for computed tomography and color Doppler ultrasonography (CDUS), with the objective of identifying cases of PE and DVT in these patients. The present review found that CDUS was the diagnostic method most used to identify DVT in the lower limbs of patients with COVID-19. Early diagnosis of DVT in the lower limbs of patients with COVID-19 is of fundamental importance for good prognosis. It should be stressed that examinations to investigate vascular changes are important regardless of the presence of signs or symptoms of DVT, especially in patients with severe cases of COVID-19, admitted to intensive care units, and/or on ventilatory support, with the purpose of diagnosis and monitoring of the problem, considering that the prevalence of DVT in lower limbs was higher in more severe cases and in the presence of immobilization.^50^

Analysis of the treatments employed for management of DVT in the lower limbs of patients with COVID-19 suggests that early administration of prophylactic anticoagulant is beneficial to the prognosis of critical patients with COVID-19 pneumonia and will probably reduce the rates of thromboembolic events. However, there are inconsistencies in the literature.^14,28,30,41,44,45^

The studies included in this review describe patients with COVID-19 of varying degrees of severity and from different population groups. The lack of additional detailed information about the health conditions of these patients was a factor that made in-depth analysis of these issues difficult, which can be considered a limitation of the study. Moreover, the analysis conducted in this review should be updated constantly, considering that different periods of time since the disease emerged may yield different findings. It is clear that further studies are needed to confirm the applicability and effectiveness in specific population groups of the diagnostic methods and treatments used to treat DVT in the lower limbs of patients with COVID-19. The disease’s extreme transmissibility and lethality meant that scientific efforts and resources were initially directed towards discovering preventative methods, with emphasis on development of vaccines for SARS-CoV-2, while the disease’s multisystemic characteristics are now being better understood.

## CONCLUSIONS

There was major variability in the prevalence of DVT in patients with COVID-19 and DVT in the lower limbs appears to be associated with more severe cases of COVID-19, such as in patients admitted to intensive care units with respiratory distress with respiratory rate ≥ 30 breaths/minute; peripheral oxy-hemoglobin saturation ≤ 93% at rest; ratio of partial pressure of oxygen/fraction of inspired oxygen ≤ 300 mmHg; respiratory failure requiring mechanical ventilation; shock; or failure of other organs.
