# Supplementary material for: Deep vein thrombosis of lower limbs in patients with COVID-19
Source: J Vasc Bras. 2023 Nov 27;22:e20230027. doi: 10.1590/1677-5449.202300272 (PMC10706010; doi:10.1590/1677-5449.202300272)
Supplement: Translation

# Translation: Trombose venosa profunda de membros inferiores em pacientes com covid-19

**DOI:** 10.1590/1677-5449.202300271

**Authors:** Orlando Adas Saliba, Ana Flávia de Jesus Alves, Camila Matarazzo, Gabriela Teixeira Gonçalves, Marcone Lima Sobreira

**Affiliations:** 1 Universidade Estadual Paulista “Júlio de Mesquita Filho” - UNESP, Botucatu, SP, Brasil.; 2 Centro Universitário Católico Auxilium - UniSALESIANO, Araçatuba, SP, Brasil.

**Keywords:** anticoagulante, SARS-CoV-2, tromboembolia

## Abstract

Com o avanço do conhecimento, a covid-19 passou a ser considerada uma doença do sistema respiratório, podendo ter comprometimento multissistêmico. Analisou-se a prevalência de trombose venosa profunda (TVP) em membros inferiores em pacientes acometidos pela covid-19 através de uma pesquisa de revisão integrativa, considerando o período de 2019 a 2022. Os procedimentos utilizados para a seleção dos artigos foram identificação das palavras-chave, elaboração da estratégia de busca, consulta em bases de dados e exclusão dos artigos em duplicata e outros. A exclusão foi feita com base nos seguintes critérios: artigos sobre complicações vasculares arteriais em membros inferiores, pesquisas laboratoriais, relatos de casos referentes a complicações venosas e arteriais em outros sítios e artigos não relacionados ao desfecho de TVP. Do total de 284 artigos, foram incluídos 42. Observou-se grande variabilidade na prevalência de TVP em pacientes com covid-19 (0,43 a 60,87%). Sugere-se que a ocorrência de TVP em pacientes com covid-19 está associada à gravidade desta doença.

## INTRODUÇÃO

Os coronavírus (CoVs) são vírus de ácido ribonucleico (RNA) de fita simples que causam doenças em humanos e animais^1^ . Em dezembro de 2019, foi descoberta uma nova cepa de coronavírus em pacientes que apresentavam uma pneumonia de etiologia desconhecida, nomeado coronavírus 2 da síndrome respiratória aguda grave (SARS-CoV-2) pelo Comitê Internacional de Taxonomia de Vírus (ICTV)^1^ . Nesse mesmo mês, foi confirmado o primeiro caso da doença do coronavírus 2019 (covid-19) na cidade de Wuhan, na China^2^ . Essa doença gerou um surto global que, em 11 de março de 2020, foi considerada uma pandemia pela Organização Mundial da Saúde (OMS)^3^ .

A pandemia teve uma rápida progressão, tendo sido registrados mais de 199 milhões de casos confirmados e mais de 4 milhões de mortes ao longo de 2021 mundialmente^4^ , demonstrando altas taxas de contágio, morbimortalidade e letalidade, o que gerou uma grande sobrecarga para os sistemas de saúde^5^ .

A transmissão da covid-19 ocorre de pessoa para pessoa, por meio de contato direto, por transmissões aéreas por meio de aerossóis e durante procedimentos médicos^6^ . O período de incubação do SARS-CoV-2 pode ser considerado entre 2 e 14 dias, com média de 5 dias^7^ , e o diagnóstico é realizado através do exame de *swab* nasofaríngeo com reação de transcriptase reversa seguida de reação em cadeia da polimerase (RT-PCR)^8^ .

Cerca de 80% dos casos confirmados de covid-19 são assintomáticos ou sintomáticos leves^9^ ; 15% apresentam uma forma mais grave; e 5% desenvolvem síndrome da angústia respiratória aguda (SARA) e necessitam de suporte ventilatório^7^ .

Os pacientes considerados com gravidade de leve a moderada apresentam as seguintes manifestações clínicas: febre (88,7%), tosse (67,8%), fadiga (38,1%), produção de expectoração (33,4%), dispneia (18,7%), dor de garganta (13,9%) e dor de cabeça (13,6%)^1^ .

A infecção grave é caracterizada por resposta imunológica e inflamatória multissistêmica descontrolada, com comprometimento cardiovascular, respiratório, neurológico, intestinal, hepático, pancreático, renal, cutâneo e hematológico. Essa resposta é mediada por ação direta do vírus, com disfunção endotelial, inflamação e trombose da microcirculação dos órgãos^10^ , podendo ocorrer sepse, SARA, pneumonia intersticial bilateral, falência múltipla de órgão e coagulação intravascular disseminada (CID), cujo curso pode ser fatal^11^ . Essa ativação da coagulação sanguínea gera um estado pró-trombótico com elevação significativa de fibrina, de produtos de degradação de fibrina (incluindo o dímero-D [DD]) e fibrinogênio. Esse estado é denominado coagulopatia associada à covid-19 (CAC). A fisiopatologia da CAC inclui uma tempestade de citocinas, causando ativação das células endoteliais e lesão na microvasculatura pulmonar que leva à microtrombose local e a um estado de hipercoagulabilidade que pode causar trombose de grandes vasos^2^ .

Nesta perspectiva, a natureza pró-coagulante da covid-19 associada a fatores de risco, como imobilidade, ventilação mecânica e infecção, predispõem o paciente a complicações tromboembólicas^7^ , como trombose venosa profunda (TVP), embolia pulmonar (EP), trombose arterial, trombose pulmonar (TP)^12^ , trombose incomum de linhas centrais ou cateteres arteriais, tromboses precoces de filtros de hemodiálise extrarrenal e cânulas de oxigenação por membrana extracorpórea (ECMO)^7^ .

Considerando a importância do acometimento vascular relacionado à covid-19 e da necessidade de dados para tomada de decisão sobre as formas mais eficazes de tromboprofilaxia, é necessário discutir o impacto dessas complicações, assim como as diferentes formas de abordagem terapêutica, justificando uma busca ampla na literatura.

Diante do exposto, o objetivo deste estudo foi analisar a prevalência de TVP em membros inferiores de pacientes acometidos pela covid-19.

## MÉTODOS

Tratou-se de uma pesquisa de revisão integrativa de literatura sobre TVP em membros inferiores relacionada à covid-19, escrita de acordo com o protocolo PRISMA^13^ .

Quanto aos procedimentos empregados para o levantamento e a seleção da produção científica, foram consultadas as bases de dados SciELO, PubMed, Cochrane, Scopus, Web of Science e LILACS, considerando o período de dezembro de 2019, início da pandemia, a setembro de 2022.

Foram selecionadas as palavras-chave, sendo definida a seguinte estratégia de busca: COVID19 or Doença Viral COVID-19 or Doença por 2019-nCoV or Doença por Coronavírus 2019 or Doença por Coronavírus 2019-nCoV or Doença por Coronavírus-19 or Doença por Novo Coronavírus de 2019 or Doença por Vírus COVID-19 or Epidemia de Pneumonia por Coronavírus de Wuhan or Epidemia de Pneumonia por Coronavírus de Wuhan de 2019-2020 or Epidemia de Pneumonia por Coronavírus em Wuhan or Epidemia de Pneumonia por Coronavírus em Wuhan de 2019-2020 or Epidemia de Pneumonia por Novo Coronavírus de 2019-2020 or Epidemia pelo Coronavírus de Wuhan or Epidemia pelo Coronavírus em Wuhan or Epidemia pelo Novo Coronavírus 2019 or Epidemia por 2019-nCoV or Epidemia por Coronavírus de Wuhan or Epidemia por Coronavírus em Wuhan or Epidemia por Novo Coronavírus 2019 or Febre de Pneumonia por Coronavírus de Wuhan or Infecção Viral COVID-19 or Infecção pelo Coronavírus 2019-nCoV or Infecção pelo Coronavírus de Wuhan or Infecção pelo SARS-CoV-2 or Infecção por 2019-nCoV or Infecção por Coronavírus 2019-nCoV or Infecção por Coronavírus de Wuhan or Infecção por Novo Coronavírus de 2019 or Infecção por SARS Coronavírus 2 or Infecção por SARS-CoV-2 or Infecção por Vírus COVID-19 or Infecções por SARS-CoV-2 or Pandemia COVID-19 or Pandemia por COVID-19 or Pandemias por COVID-19 or Pneumonia do Mercado de Frutos do Mar de Wuhan or Pneumonia por Coronavírus de Wuhan or Pneumonia por Novo Coronavírus de 2019-2020 or Surto de Coronavírus de Wuhan or Surto de Pneumonia da China 2019-2020 or Surto de Pneumonia na China 2019-2020 or Surto pelo Coronavírus 2019-nCoV or Surto pelo Coronavírus de Wuhan or Surto pelo Coronavírus de Wuhan de 2019-2020 or Surto pelo Novo Coronavírus 2019 or Surto por 2019-nCoV or Surto por Coronavírus 2019-nCoV or Surto por Coronavírus de Wuhan or Surto por Coronavírus de Wuhan de 2019-2020 or Surto por Novo Coronavírus 2019 or Virose COVID-19) and (Trombose Venosa Profunda membros inferiores or Trombose Venosa Profunda membro inferior or Trombose de Veia Profunda membros inferiores or Trombose de Veia Profunda membro inferior or Trombose de Veias Profundas membros inferiores or Trombose de Veias Profundas membro inferior or Deep Vein Thrombosis lower limbs or Deep Vein Thrombosis lower limb.

Foram incluídos estudos de coorte, de caso-controle e transversais que estivessem diretamente relacionados à temática de interesse. A análise dos artigos foi iniciada pela leitura dos títulos e resumos para classificação do tipo de pesquisa e eliminação das publicações repetidas. A seguir, realizou-se a leitura dos artigos na íntegra, com o intuito de identificar características, objetivos e resultados de cada produção. Os dados de cada artigo foram extraídos e registrados em planilha específica, na qual constava título, autores, ano da publicação, país onde foi conduzido o estudo, periódico, objetivo, tipo de estudo, amostra, exame utilizado para o diagnóstico, principais resultados e nível de evidência. Foi feita a análise para avaliar o grau de evidência dos artigos por meio do protocolo SORT (Strength of Recommendation Taxonomy). De acordo com essa classificação, os níveis de evidência são estratificados em: A) evidência consistente e de boa qualidade orientada para o paciente; B) evidência inconsistente ou de qualidade limitada orientada para o paciente; ou C) consenso, evidência orientada para a doença, prática usual, opinião de especialistas ou série de casos para estudos de diagnóstico, tratamento, prevenção ou triagem.

Foram incluídos na pesquisa os artigos relacionados ao desfecho principal considerado, TVP sintomática e assintomática, bem como casos diagnosticados incidentalmente ou de forma sistemática em membros inferiores de pacientes confirmados para covid-19. Foram excluídos os artigos do tipo relatos de caso e cuja temática era sobre complicações vasculares arteriais e venosas localizadas em outros sítios anatômicos. Do total de 284 artigos encontrados, 42 foram analisados.

A Figura 1 demonstra os procedimentos adotados e o processo de seleção dos artigos nas bases de dados.

**Figura 1:** Estratégia de busca usada para revisão da literatura sobre trombose venosa profunda de membros inferiores em pacientes com covid-19.

## RESULTADOS

A Tabela 1 apresenta as principais informações extraídas de cada um dos 42 artigos científicos selecionados, incluindo título, autores, ano da publicação, país onde foi conduzido o estudo, periódico, objetivo, tipo de estudo, amostra, exame utilizado para o diagnóstico da TVP e principais resultados sobre a prevalência da doença. Os estudos incluídos nesta revisão (n = 42) foram realizados na Itália (n = 12), Estados Unidos (n = 8), França (n = 6), China (n = 5), Espanha (n = 4), Brasil (n = 2), Argentina (n = 1), Croácia (n = 1), Egito (n = 1), Suécia (n = 1) e Suíça (n = 1). Nota-se que a maioria dos artigos publicados foi estudos do tipo coorte. O exame complementar mais empregado para o diagnóstico de TVP em membros inferiores foi o ultrassom. Houve grande variabilidade nos resultados de prevalência (0,43 a 60,87%). Nota-se que grande parte dos artigos foi classificada com nível de evidência A (n = 19).

**Tabela 1:** Estudos selecionados sobre trombose venosa profunda em membros inferiores de pacientes acometidos por covid-19

**N.º**	**Título**	**Autor**	**Ano**	**País**	**Revista**	**Objetivo**	**Tipo de estudo**	**Tamanho da amostra**	**Exame**	**Resultados** **(número de pacientes com TVP)**	**Nível de evidência**
1	Thrombosis and bleeding after implementation of an intermediate-dose prophylactic anticoagulation protocol in ICU patients with COVID-19: a multicenter screening study	Al-Abani et al.^14^	2022	Suécia	Journal of Intensive Care Medicine	Estudar a prevalência de tromboembolismo venoso e sangramento em pacientes com quadro crítico de covid-19 expostos a dose intermediária de tromboprofilaxia.	Estudo observacional prospectivo	100 pacientes	Ultrassom com manobra de compressão em duas regiões das extremidades inferiores, incluindo as veias femoral comum, femoral e poplítea, com o paciente na posição supina.	20/100 pacientes (20,00%), sendo 19 casos assintomáticos	A
2	Venous Doppler ultrasound in critically ill COVID-19 patients: game changer in anticoagulation therapy	Alfageme et al.^15^	2020	Espanha	Ultrasound Journal	Avaliar a prevalência de trombose venosa profunda assintomática em membros inferiores em pacientes criticamente enfermos com covid-19, com insuficiência respiratória grave e altos níveis de dímero-D.	Estudo observacional, descritivo e retrospectivo	23 pacientes	Ultrassom Doppler	14/23 pacientes (60,87%), sendo 5 em território venoso proximal e 9 em veias infrapoplíteas	C
3	COVID-19 and venous thromboembolism in intensive care or medical ward	Avruscio et al.^16^	2020	Itália	Clinical and Translational Science	Avaliar a incidência de tromboembolismo venoso em pacientes com covid-19 internados em unidades de terapia intensiva e enfermarias médicas.	Estudo observacional de coorte	85 pacientes, sendo 44 internados em enfermarias médicas e 41, em unidades de terapia intensiva	Ultrassonografia com Doppler colorido bilateral sistemática	20/85 pacientes (23,53%), sendo 6 pacientes internados em enfermarias médicas e 14, em unidades de terapia intensiva	B
4	Incidence of deep venous thrombosis in COVID-19 hospitalized patients during the first peak of the Italian outbreak	Baccellieri et al.^17^	2021	Itália	Phlebology	Avaliar a incidência de trombose venosa profunda em pacientes com covid-19 internados em um hospital terciário.	Estudo observacional retrospectivo	200 pacientes	Ultrassonografia vascular em todos os pacientes	29/200 pacientes (14,50%), sendo 5/40 (12,50%) em pacientes de unidade de terapia intensiva e 24/160 (15,00%) em pacientes não internados na unidade de terapia intensiva	B
5	Deep vein thrombosis in hospitalized patients with coronavirus disease 2019	Chang et al.^18^	2021	Estados Unidos	Journal of Vascular Surgery: Venous and Lymphatic Disorder	Avaliar a experiência com trombose venosa profunda em pacientes com covid-19.	Estudo de análise de coorte retrospectiva de centro único	188 pacientes	Ultrassom *duplex* realizado por tecnólogos vasculares credenciados e revisados e formalmente lido por cirurgiões vasculares	53/188 pacientes (28,20%).	C
6	Pulmonary embolism or thrombosis in ARDS COVID-19 patients: a French monocenter retrospective study	Contou et al.^19^	2020	França	PLoS One	Avaliar as características clínicas e os desfechos dos pacientes com síndrome do desconforto respiratório agudo causada por covid-19, diagnosticados com embolia pulmonar durante a internação na unidade de terapia intensiva.	Estudo observacional retrospectivo	16 pacientes	Ultrassonografia Doppler venosa de membros inferiores dos pacientes que apresentaram embolia pulmonar	3/16 pacientes (19,00%)	A
7	Comparison of deep vein thrombosis risks in acute respiratory distress syndrome caused by COVID-19 and bacterial pneumonia: a retrospective cohort study	Cui et al.^20^	2022	China	Thrombosis Journal	Comparar a incidência e os riscos de trombose venosa profunda entre pacientes com síndrome do desconforto respiratório agudo causada por covid-19 e por pneumonia bacteriana.	Estudo de coorte retrospectivo	105 pacientes	Ultrassonografia com manobra de compressão venosa, nos membros inferiores, incluindo as veias femorais comuns bilaterais, femorais profundas e superficiais, as veias poplíteas e as veias tibial anterior, tibial posterior, fibular e da panturrilha	60/105 pacientes (57,10%)	B
8	Is venous thromboembolism a predictable marker in older patients with COVID-19 infection? A single-center observational study	De Giorgi et al.^21^	2021	Itália	The Journal of Infection in Developing Countries	Analisar se exames laboratoriais, escores de avaliação de risco e escores de comorbidade foram úteis na previsão de trombose venosa profunda em pacientes com SARS-CoV-2 admitidos na ala de medicina interna.	Estudo observacional de coorte	49 pacientes	Ultrassonografia venosa com Doppler colorido dos membros inferiores	10/49 pacientes (20,40%)	A
9	Imaging of COVID-19 vasculopathy from head to toe: Egyptian collective experience after 2 years of the pandemic	Fathy et al.^22^	2022	Egito	Egyptian Journal of Radiology and Nuclear Medicine	Relatar a experiência egípcia sobre a vasculopatia por covid-19 e incluir coletivamente as diferentes modalidades e técnicas de imagem para o diagnóstico de complicações vasculares cerebrovasculares, pulmonares, gastrointestinais e arteriais periféricas.	Estudo de coorte retrospectivo	282 pacientes	Exames de ultrassom *duplex*	39/282 pacientes (13,80%)	A
10	COVID-19: persistence of symptoms and lung alterations after 3-6 months from hospital discharge	Fortini et al.^23^	2021	Itália	Infection	Avaliar os resultados de um programa de acompanhamento para pacientes que receberam alta de uma enfermaria não intensiva de covid-19.	Estudo observacional prospectivo	59 pacientes	Ultrassonografia com manobra de compressão	1/59 pacientes (1,70%)	A
11	Point of care ultrasound (POCUS) in diagnosis of proximal deep vein thrombosis among COVID-19 hospitalized patients with a high rate of low molecular weight heparin prophylaxis	García-Ceberino et al.^12^	2021	Espanha	Medicina Clinica	Determinar a prevalência de trombose venosa profunda proximal com rastreamento por ultrassom no local de atendimento entre pacientes hospitalizados com covid-19.	Estudo observacional retrospectivo	87 pacientes	Ultrassonografia de membros inferiores	4/87 pacientes (4,60%)	C
12	Probative value of the D-dimer assay for diagnosis of deep venous thrombosis in the coronavirus disease 2019 syndrome	Gibson et al.^24^	2020	Estados Unidos	Critical Care Medicine	Descrever a utilidade preditiva do ensaio de dímero-D entre pacientes com a síndrome da doença de coronavírus 2019 para trombose venosa profunda não provocada dos membros inferiores.	Estudo observacional prospectivo	72 pacientes	Ultrassonografia *duplex* de membros inferiores	12/72 pacientes (16,70%)	A
13	SARS-CoV-2 and finding of vein thrombosis: can IMPROVE and IMPROVEDD scores predict COVID-19 outcomes?	Greco et al.^25^	2021	Itália	European Review for Medical and Pharmacological Sciences	Analisar a relação entre escores preditivos validados para doenças tromboembólicas venosas, como IMPROVE e IMPROVEDD, e a intensificação de cuidados, taxa de mortalidade intra-hospitalar e taxa de mortalidade em 30 dias.	Estudo observacional retrospectivo	51 pacientes	Ultrassonografia venosa com Doppler colorido de membros inferiores	11/51 pacientes (21,60%)	A
14	Systematic duplex ultrasound screening in conventional units for COVID-19 patients with follow-up of 5 days	Hamadé et al.^26^	2021	França	Journal of Vascular Surgery: Venous and Lymphatic Disorders	Avaliar a prevalência de trombose venosa profunda de membros inferiores por meio de ultrassonografia em pacientes infectados por covid-19.	Estudo de coorte retrospectivo	72 pacientes	Ultrassom *duplex* venoso de membros inferiores	12/72 pacientes (16,70%)	C
15	Efficacy and safety of sonographer discretion to terminate a venous duplex ultrasound for diagnosis of deep vein thrombosis in coronavirus disease 2019 patients	Ho et al.^27^	2022	Estados Unidos	Journal of Vascular Surgery: Venous and Lymphatic Disorders	Avaliar a eficácia de um protocolo de ultrassom *duplex* venoso modificado para reduzir a exposição do ultrassonografista a pacientes com covid-19.	Estudo de coorte retrospectivo	168 pacientes	Ultrassom *duplex* venoso de membros inferiores	44/168 pacientes (27,50%)	A
16	Deep venous thrombosis incidence in patients with COVID-19 acute respiratory distress syndrome, under intermediate dose of chemical thromboprophylaxis	Hunter et al.^28^	2022	Argentina	Medicina (Buenos Aires)	Avaliar a incidência de trombose venosa profunda em pacientes com pneumonia grave por covid-19, com necessidade de ventilação mecânica e sob dose intermediária de tromboprofilaxia química.	Estudo de coorte prospectivo	46 pacientes	Ultrassonografia Doppler de membros inferiores	3/46 pacientes (6,52%)	A
17	Deep vein thrombosis in COVID-19 patients in general wards: prevalence and association with clinical and laboratory variables	Ierardi et al.^29^	2021	Itália	La Radiologia Medica	Relatar a prevalência de trombose venosa profunda em membros inferiores de pacientes com covid-19 em enfermarias gerais.	Estudo transversal	263 pacientes	Ultrassonografia *duplex* de membros inferiores	67/263 pacientes (25,50%)	C
18	Examining D-dimer and empiric anti-coagulation in COVID-19-related thrombosis	Johnson et al.^30^	2022	Estados Unidos	The Cureus Journal of Medical Science	Elucidar a relação entre trombose macro/microvascular, níveis de dímero-D e anticoagulação empírica na covid-19.	Estudo observacional prospectivo	52 pacientes	Ultrassonografia *duplex* venosa de extremidades inferiores	1/52 pacientes (1,9%)	A
19	Thromboembolic events and role of point of care ultrasound in hospitalized Covid-19 patients needing intensive care unit admission	Kapoor et al.^2^	2021	Estados Unidos	Journal of Intensive Care Medicine	Investigar a incidência de eventos tromboembólicos em pacientes com covid-19 em unidade de terapia intensiva e avaliar a utilidade do ultrassom no local de atendimento para diagnosticar trombose venosa profunda em membros inferiores.	Estudo observacional prospectivo	107 pacientes	Ultrassonografia de membros inferiores	21/107 pacientes (19,63%)	C
20	Screening for deep vein thrombosis in persons with COVID-19 upon admission to an inpatient rehabilitation hospital	Kirshblum et al.^3^	2021	Estados Unidos	American Journal of Physical Medicine & Rehabilitation	Determinar a prevalência de trombose venosa profunda detectada por triagem *duplex* e os fatores de risco associados à trombose venosa profunda em pacientes com covid-19.	Estudo observacional retrospectivo	113 pacientes	Ultrassonografia *duplex* de triagem de ambos os membros inferiores	25/113 pacientes (22,00%)	A
21	Clinical characteristics of acute lower extremity deep venous thrombosis diagnosed by duplex in patients hospitalized for coronavirus disease 2019	Koleilat et al.^31^	2021	Estados Unidos	Journal of Vascular Surgery: Venous and Lymphatic Disorders	Caracterizar pacientes com trombose venosa profunda identificada após a admissão por covid-19.	Estudo retrospectivo de caso-controle	135 pacientes	Ultrassonografia *duplex* venosa de extremidades inferiores	18/135 pacientes (13,30%)	C
22	Systematic screening for deep vein thrombosis in critically ill in patients with COVID-19: impact on the incidence of venous thromboembolism	Lapébie et al.^32^	2021	França	Frontiers in Medicine	Comparar a incidência de tromboembolismo venoso por meio de dois métodos para diagnóstico de trombose venosa profunda de membros inferiores.	Estudo de coorte prospectivo	27 pacientes	Ultrassonografia venosa com manobra de compressão completa dos membros inferiores	7/27 pacientes (25,90%)	B
23	Venous thromboembolism in critically Ill patients with COVID-19: results of a screening study for deep vein thrombosis	Longchamp et al.^33^	2020	Suíça	Research and Practice in Thrombosis and Haemostasis	Determinar a prevalência de tromboembolismo venoso em pacientes críticos com covid-19, usando triagem ultrassonográfica venosa de membros inferiores.	Estudo observacional prospectivo	25 pacientes	Ultrassom com manobra de compressão venosa de membros inferiores	6/25 pacientes (24,00%)	C
24	Asymptomatic deep vein thromboses in prolonged hospitalized COVID-19 patients	Lucijanic et al.^34^	2021	Croácia	Wiener klinische Wochenschrift	Avaliar a prevalência de trombose venosa profunda entre pacientes com covid-19 hospitalizados por tempo prolongado, sem suspeita clínica de trombose venosa profunda, e investigar possíveis preditores.	Estudo observacional prospectivo	102 pacientes	Ultrassonografia *duplex* bilateral de veias profundas de membros inferiores	26/102 pacientes (25,50%)	A
25	Pulmonary thromboembolism in hospitalised COVID-19 patients at moderate to high risk by Wells score: a report from Lombardy, Italy	Monfardini et al.^35^	2020	Itália	The British Journal of Radiology	Relatar a experiência de um centro de angiografia pulmonar na avaliação de pacientes hospitalizados com covid-19 com risco moderado a alto de tromboembolismo pulmonar.	Estudo observacional retrospectivo	33 pacientes	Ultrassonografia Doppler venosa de membros inferiores	4/33 pacientes (12,12%)	C
26	Lower limb deep vein thrombosis in COVID-19 patients admitted to intermediate care respiratory units	Pancani et al.^36^	2021	Itália	Thrombosis Research	Investigar a incidência de trombose venosa profunda de membros inferiores em pacientes com covid-19 admitidos em enfermarias médicas e que não necessitavam de unidade de terapia intensiva.	Estudo observacional prospectivo	66 pacientes	Ultrassonografia com manobra de compressão	2/66 pacientes (3,00%)	C
27	Incidence of venous thromboembolism in patients with non-hematological cancer admitted for COVID-19 at a third-level hospital in Madrid	Paredes-Ruiz et al.^37^	2022	Espanha	Journal of Thrombosis and Thrombolysi	Descrever a taxa de incidência de tromboembolismo venoso em pacientes com câncer não hematológico que necessitaram de internação por covid-19.	Estudo observacional prospectivo	11 pacientes	Ultrassonografia Doppler de membros inferiores	2/11 pacientes (18,00%)	C
28	Mortality and change in the prevalence of deep vein thrombosis associated with SARS-CoV-2 P.1 variant	Pereira de Godoy et al.^5^	2022	Brasil	Cureus	Determinar as taxas de mortalidade, mensal e geral, por sexo e faixa etária em pacientes hospitalizados por covid-19 e a prevalência de trombose venosa profunda nesses pacientes.	Estudo de caso-controle	6.199 pacientes	Ultrassonografia eco-Doppler venosa bilateral	254/6.199 pacientes (4,10%)	A
29	Incidence of deep vein thrombosis through an ultrasound surveillance protocol in patients with COVID-19 pneumonia in non-ICU setting: a multicenter prospective study	Pieralli et al.^38^	2021	Itália	PLoS One	Avaliar a incidência de trombose venosa profunda dos membros inferiores em pacientes agudamente enfermos com pneumonia por covid-19, admitidos em unidades de terapia não intensiva.	Estudo de coorte prospectivo	227 pacientes	Doppler codificado por cores seriado e ultrassonografia com manobra de compressão	31/277 pacientes (13,70%)	C
30	Coagulation parameters and venous thromboembolism in patients with and without COVID-19 admitted to the Emergency Department for acute respiratory insufficiency	Pizzi et al.^39^	2020	Itália	Thrombosis Research	Comparar os parâmetros de coagulação na admissão entre pacientes com covid-19 e sem covid-19 com insuficiência respiratória aguda e descrever os eventos tromboembólicos venosos diagnosticados.	Estudo observacional retrospectivo	162 pacientes	Ultrassonografia com manobra de compressão limitada dos membros inferiores	3/162 pacientes (1,85%)	A
31	Combined use of Wells scores and D-dimer levels for the diagnosis of deep vein thrombosis and pulmonary embolism in COVID-19: a retrospective cohort study	Raj et al.^40^	2021	Estados Unidos	Cureus	Avaliar a utilidade dos escores de trombose venosa profunda, de embolia pulmonar de Wells e de dímero-D no diagnóstico de trombose venosa profunda e embolia pulmonar em pacientes com covid-19.	Estudo de coorte retrospectivo	106 pacientes	*Duplex* de extremidade inferior	35/106 pacientes (33,00%)	A
32	Rivaroxaban versus no anticoagulation for post-discharge thromboprophylaxis after hospitalisation for COVID-19 (MICHELLE): an open-label, multicentre, randomised, controlled trial	Ramacciotti et al.^41^	2022	Brasil	Lancet	Verificar se pacientes hospitalizados com covid-19 que receberam a profilaxia com rivaroxabana (10 mg/dia por 35 dias após a alta) apresentam melhora em desfechos clínicos, incluindo eventos tromboembólicos maiores e fatais.	Estudo clínico multicêntrico, randomizado e controlado	252 pacientes	Ultrassom Doppler venoso bilateral de membros inferiores	4/252 pacientes (1,59%)	A
33	Extremely high incidence of lower extremity deep venous thrombosis in 48 patients with severe COVID-19 in Wuhan	Ren et al.^42^	2020	China	Circulation	Analisar a prevalência da formação de trombose em pacientes com covid-19.	Estudo transversal	48 pacientes	Ultrassom com manobra de compressão nas extremidades inferiores	41/48 pacientes (85,40%)	C
34	High prevalence of deep venous thrombosis in non-severe COVID-19 patients hospitalized for a neurovascular disease	Rouyer et al.^43^	2020	França	Cerebrovascular Diseases Extra	Relatar os dados obtidos após a ultrassonografia com Doppler sistemática em membros inferiores de pacientes com covid-19 não grave.	Estudo observacional prospectivo	13 pacientes	Ultrassonografia Doppler nos membros inferiores	5/13 pacientes (38,46%)	C
35	Incidence of deep vein thrombosis among non-ICU patients hospitalized for COVID-19 despite pharmacological thromboprophylaxis	Santoliquido et al.^44^	2020	Itália	Journal of Thrombosis and Haemostasis	Determinar a incidência de tromboembolismo venoso em pacientes não internados em unidade de terapia intensiva por covid-19, que receberam tromboprofilaxia farmacológica.	Estudo de coorte retrospectivo	84 pacientes	Ultrassonografia com manobra de compressão venosa nos membros inferiores	10/84 pacientes (11,90%)	A
36	Pulmonary thromboembolism in coronavirus disease 2019 patients undergoing thromboprophylaxis	Schiaffino et al.^45^	2021	Itália	Medicine (Baltimore)	Investigar a prevalência de tromboembolismo pulmonar e sua associação com variáveis clínicas em uma coorte de pacientes hospitalizados com covid-19, recebendo heparina de baixo peso molecular em dosagem profilática.	Estudo observacional retrospectivo	33 pacientes	Ultrassonografia Doppler nos membros inferiores	1/33 pacientes (3,00%)	A
37	Duplex ultrasound screening for deep and superficial vein thrombosis in COVID-19 patients	Tung-Chen et al.^46^	2022	Espanha	Journal of Ultrasound in Medicine	Determinar a real incidência de trombose venosa profunda ou superficial em pacientes com covid-19.	Estudo observacional prospectivo	233 pacientes	Ultrassonografia *duplex* bilateral de membros inferiores	1/233 pacientes (0,43%)	C
38	Characteristics of deep vein thrombosis in the critically ill COVID-19 patient - an observational cohort study with Doppler ultrasound measurements	Voicu et al.^47^	2022	França	European Review for Medical and Pharmacological Sciences	Relatar as características da trombose venosa profunda, os diâmetros das veias e as velocidades de pico do fluxo sanguíneo nas veias femorais comuns de pacientes criticamente enfermos com covid-19.	Estudo de coorte prospectivo	55 pacientes	Ultrassonografia Doppler de membros inferiores	19/55 pacientes (35,00%)	C
39	Imbalance between procoagulant factors and natural coagulation inhibitors contributes to hypercoagulability in the critically ill COVID-19 patient: clinical implications	Voicu et al.^48^	2020	França	European Review for Medical and Pharmacological Sciences	Investigar o equilíbrio entre fatores pró-coagulantes e inibidores naturais da coagulação no paciente grave com covid-19 e avaliar a utilidade dos parâmetros de hemostasia para identificar pacientes com risco de evento tromboembólico venoso.	Estudo observacional prospectivo	85 pacientes	Ultrassom *duplex* venoso dos membros inferiores	35/85 pacientes (41,18%)	A
40	Analysis of risk factors for thromboembolic events in 88 patients with COVID-19 pneumonia in Wuhan, China: a retrospective descriptive report	Wang et al.^49^	2021	China	Medical Science Monitor	Analisar os fatores de risco e a incidência de trombose em pacientes com pneumonia confirmada por covid-19.	Estudo observacional retrospectivo	88 pacientes	Ultrassom Doppler	19/88 pacientes (21,59%)	B
41	Incidence and risk factors of deep vein thrombosis in hospitalized COVID-19 patients	Yu et al.^50^	2020	China	Clinical and Applied Thrombosis/Hemostasis	Avaliar a taxa de incidência e os fatores de risco de trombose venosa profunda em pacientes com covid-19.	Estudo observacional retrospectivo	142 pacientes	Ultrassom com manobra de compressão venosa de membros inferiores	50/142 pacientes (35,21%)	C
42	Deep vein thrombosis in hospitalized patients with COVID-19 in Wuhan, China: prevalence, risk factors, and outcome	Zhang et al.^51^	2020	China	Circulation	Investigar trombose venosa profunda em pacientes hospitalizados com covid-19.	Estudo observacional transversal	143 pacientes	Ultrassonografia venosa de membros inferiores	66/143 pacientes (46,15%)	C

## DISCUSSÃO

Nesta pesquisa de revisão integrativa, verificou-se que vários estudos têm sido realizados em diferentes regiões do mundo com o intuito de investigar sinais e sintomas da covid-19, bem como complicações sistêmicas, como a TVP de membros inferiores. Os dados clínicos e epidemiológicos são fundamentais para aprimorar o planejamento e a execução dos cuidados assistenciais nos serviços de saúde disponibilizados aos pacientes acometidos pelas doenças.

Nota-se que a maioria das pesquisas foram desenvolvidas em países dos continentes europeu e americano, enquanto apenas um estudo foi conduzido no continente africano. Neste contexto, evidencia-se a importância da realização de estudos com diferentes populações, considerando a diversidade de etnias, culturas, condições sociais e econômicas, com o objetivo de melhor compreender a característica multissistêmica da doença e identificar possíveis fatores que podem estar associados à ocorrência de TVP de membros inferiores em pacientes acometidos pela covid-19.

Na presente pesquisa, verificou-se que a incidência de casos de TVP de membros inferiores em pacientes com covid-19 foi investigada em diferentes grupos populacionais, com níveis leve, moderado e grave de acometimento pela covid-19, de modo que é possível que esses fatores tenham influenciado a variabilidade de informações reportadas pelos estudos analisados.

Em pesquisa realizada no Union Hospital de Wuhan, na China, com 88 pacientes internados com pneumonia confirmada por covid-19, sendo 31 casos críticos, 33 casos graves e 24 casos leves, verificou-se a ocorrência de TVP em membros inferiores em 19 pacientes (21,59%), sendo 12 em casos críticos e 7 em casos graves. Assim, sugere-se que a ocorrência de TVP em pacientes com covid-19 pode estar relacionada com a gravidade da doença, sendo maior em pacientes internados em unidades de tratamento intensivo que apresentem desconforto respiratório com frequência respiratória ≥ 30 respirações/minuto; saturação periférica da oxi-hemoglobina ≤ 93% em repouso; relação pressão parcial de oxigênio/fração inspirada de oxigênio ≤ 300 mmHg; insuficiência respiratória que requer ventilação mecânica; choque; ou falência de outros órgãos^16,50^ .

É importante ressaltar que, embora os possíveis mecanismos e fatores relacionados às alterações tromboembólicas encontradas em pacientes com covid-19 não estejam totalmente elucidados, a literatura tem demonstrado que a formação de citocinas pró-inflamatórias, a indução de fatores pró-coagulantes e as alterações hemodinâmicas que predispõem à isquemia e à trombose podem contribuir para o desenvolvimento de TVP de membros inferiores nesses pacientes. Além disso, alterações detectadas por meio de exames laboratoriais complementares, como linfopenia, neutrofilia, aumento do tempo de protrombina e elevação do DD, têm sido encontradas em pacientes que apresentam tromboembolismo venoso^5^ .

Estudos recentes têm revelado que os pacientes hospitalizados devido à covid-19 possuem alto risco de desenvolvimento de eventos tromboembólicos, e, consequentemente, houve um aumento massivo no requerimento de tomografias computadorizadas e eco-Doppler colorido (EDC), com o intuito de identificar casos de EP e TVP nesses pacientes. Nesta revisão, verificou-se que o método diagnóstico mais utilizado para a identificação da TVP de membros inferiores em pacientes com covid-19 foi o EDC. O diagnóstico precoce da TVP de membros inferiores em pacientes com covid-19 é fundamental para um bom prognóstico. Convém salientar que, independentemente da presença de sinais ou sintomas de TVP, a realização de exames para investigar alterações vasculares mostra-se importante, principalmente em pacientes com casos graves de covid-19, internados em unidades de tratamento intensivo e/ou que estejam utilizando suporte ventilatório, com finalidade diagnóstica e de monitoramento da evolução do problema, considerando que a prevalência de TVP em membros inferiores foi maior em casos mais graves, em situação de imobilização^50^ .

A análise dos tratamentos empregados para o manejo da TVP de membros inferiores em pacientes com covid-19 sugere que a administração precoce de anticoagulante profilático beneficiaria o prognóstico de pacientes críticos com pneumonia por covid-19 e, provavelmente, reduziria as taxas de eventos tromboembólicos. Entretanto, há controvérsias na literatura^14,28,30,41,44,45^ .

Os estudos incluídos na pesquisa envolvem pacientes com variados níveis de gravidade da covid-19 e de grupos populacionais distintos. A falta de informações adicionais mais detalhadas sobre as condições de saúde dos pacientes foi um fator dificultador para o aprofundamento da questão, o que pode ser considerada uma limitação do estudo. Ademais, a análise realizada nesta revisão deve ser constantemente atualizada, considerando que os diferentes períodos de tempo após o surgimento da doença podem resultar em achados diferentes. Ressalta-se, portanto, a necessidade da realização de novos estudos para verificar a aplicabilidade e a efetividade dos métodos diagnósticos e terapias utilizadas no tratamento da TVP de membros inferiores em pacientes com covid-19, em grupos populacionais específicos. Reforça-se que, devido à alta transmissibilidade e letalidade da doença, os esforços e recursos científicos foram inicialmente direcionados à descoberta de métodos preventivos, com ênfase no desenvolvimento de imunizantes para o SARS-CoV-2, enquanto a característica multissistêmica da doença vem sendo melhor compreendida atualmente.

## CONCLUSÃO

Houve grande variabilidade na prevalência de TVP em pacientes com covid-19, e a TVP em membros inferiores parece estar associada a casos mais graves da covid-19, como em pacientes internados em unidades de tratamento intensivo que apresentem desconforto respiratório com frequência respiratória ≥ 30 respirações/minuto; saturação periférica da oxi-hemoglobina ≤ 93% em repouso; relação pressão parcial de oxigênio/fração inspirada de oxigênio ≤ 300 mmHg; insuficiência respiratória que requer ventilação mecânica; choque; ou falência de outros órgãos.
